# Identification and clinical implications of immune-related hub genes in psoriasis

**DOI:** 10.1371/journal.pone.0347536

**Published:** 2026-04-20

**Authors:** Yuzhen Sun, Ziguang Zhou, Yu Mao, Niu Liu, Yanfeng Li, Weiyuan Fang

**Affiliations:** 1 Department of Dermatology, The First People’s Hospital of Zhengzhou, Zhengzhou, Henan, China; 2 Department of Pulmonary and Critical Care Medicine, Huashan Hospital of Fudan University, Shanghai, China; Sukh Sagar Medical College and Hospital, INDIA

## Abstract

**Background:**

Psoriasis, a chronic inflammatory skin disease affecting 2–3% of the global population, is driven by dysregulated immune responses. Despite advancements in biologic therapies, treatment challenges persist due to high recurrence rates. This study aimed to identify immune-related hub genes and elucidate their clinical implications in psoriasis pathogenesis and therapy.

**Methods:**

Multiple microarray datasets from psoriasis patients (GSE30999, GSE106992, GSE14905, GSE78097, and GSE117468) were obtained to identify immune-key genes by differential gene analysis and Weighted Gene Co-expression Network Analysis (WGCNA). Subsequently, immune-related hub genes were identified using the Least Absolute Shrinkage and Selection Operator (LASSO) algorithm and Protein-Protein Interaction (PPI) networks, with further validation through Gene Set Enrichment Analysis (GSEA) and Receiver Operating Characteristic (ROC) curves to assess exploratory within-sample discrimination. Pearson correlation analysis evaluated the relationship between hub genes, skin lesion severity, and treatment outcomes. The study also conducted immune infiltration by using the Cell-type Identification by Estimating Relative Subsets Of RNA Transcripts (CIBERSORT) algorithm and identified potential therapeutic targets by the Drug-Gene Interaction Database (DGIdb).

**Results:**

Thirty-one immune-related key genes were identified, and six hub genes (*CLEC7A, CXCL1, IRF1, S100A12, S100A8, S100A9*) were validated as central players in immune signaling pathways. These genes exhibited within-sample discrimination (AUC > 0.9) and correlated with disease severity and biological therapy efficacy. Immune infiltration analysis revealed increased activated memory CD4^+^ T cells and M1 macrophages in lesional skin, which was strongly associated with hub gene expression. Additionally, drug-gene interaction analysis identified potential therapeutic agents targeting these genes.

**Conclusion:**

This study identified six immune-related hub genes that were closely linked to the severity of psoriasis, the effectiveness of biological treatments, and infiltrated activated memory CD4^+^ T cells and M1 macrophages. Our findings elucidate a novel immune-related hub gene network in psoriasis and provide potential targets for the development and application of biologics.

## Introduction

Psoriasis is a chronic, inflammatory, proliferative dermatological disease that affects 2−3% of the global population. It is characterized by the formation of papules and plaques on the surface of the skin, and these lesions vary in morphology, distribution, and severity [1]. While the disease predominantly manifests in two age peaks (16−22 and 55−60 years), its impact extends beyond physical symptoms, severely impairing psychosocial well-being and economic productivity [2]. Pathologically, psoriasis arises from dysregulated crosstalk between hyperproliferative keratinocytes and immune cells (e.g., Th17 cells, dendritic cells), mediated by cytokines such as IL-17 and TNF-α [3]. Current therapeutic strategies aim to control inflammation and reduce recurrence [4,5]. Despite advancements in biologic agents that modulate key cytokines such as IL-17 and TNF-α, their efficacy is still limited by interindividual variability and elevated recurrence rates [6]. This suggests the potential involvement of undiscovered critical genes within the immunoregulatory network of psoriasis.

The skin represents the initial line of defense against external insults and injuries. It contains a multitude of epidermal and immune elements that collectively constitute the skin-associated lymphoid tissue. In psoriasis, dysregulation of this immune microenvironment drives pathogenesis: activated keratinocytes release alarmins and chemokines, which recruit neutrophils and polarize macrophages toward an M1 pro-inflammatory phenotype [7,8]. These events perpetuate a feedforward loop of inflammation, mediated by IL-17 and TNF-α signaling. While biologics effectively suppress these pathways, inter-individual variability in treatment response underscores the need for biomarkers predictive of drug efficacy. Recent single-cell transcriptomic studies highlight the dynamic interplay between memory T cells and macrophage subsets in lesional skin [9,10], yet the molecular drivers linking immune cell infiltration to therapeutic resistance remain poorly defined. Notably, existing biomarker studies often rely on single-cohort analyses, with limited exploration of gene expression dynamics during biologic therapy.

Advancements in bioinformatics and multi-omics integration offer unprecedented opportunities to dissect disease mechanisms. Here, we integrated WGCNA and LASSO to systematically identify immune-related hub genes from five psoriasis datasets (GSE30999, GSE106992, GSE14905, GSE78097, GSE117468). Our objectives were threefold: (1) to validate the diagnostic and prognostic value of hub genes in independent cohorts; (2) to elucidate their association with immune cell infiltration; and (3) to explore potential therapeutic agents targeting these genes using the DGIdb. By bridging genetic drivers with clinical outcomes, this study provides novel insights into psoriasis immunopathology and identifies actionable targets for personalized therapy.

## Methods

### Data source and processing

**Fig 1:**
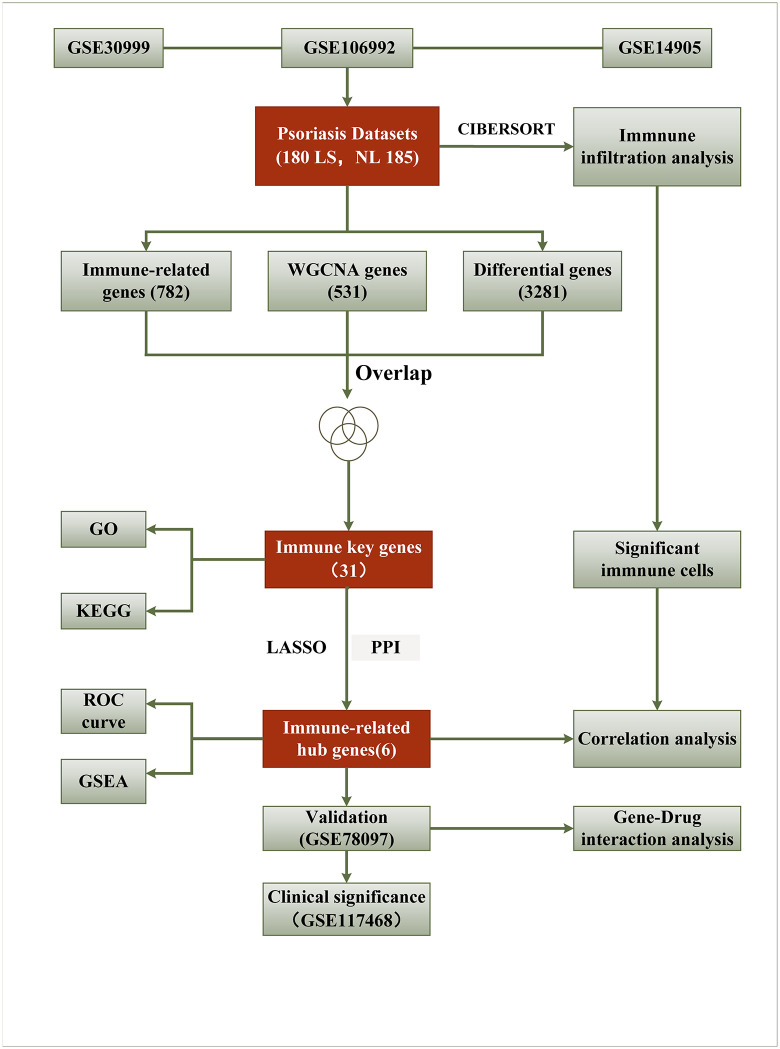
Flowchart for this study.

The workflow of this study is described as a flowchart in **Fig 1**.

As shown in **Table 1**, the microarray matrix data utilized in this study were all obtained from the Gene Expression Omnibus (GEO) database (https://www.ncbi.nlm.nih.gov/geo/). The GSE30999 [11,12], GSE106992 [13,14], and GSE14905 [15] datasets were selected as the training set, comprising 180 lesional (LS) skin samples and 185 non-lesional (NL) skin samples from patients with psoriasis, and the GSE78097 [16] dataset containing 27 LS skin samples, and 6 NL skin samples was used as the validation set. In addition, 216 LS skin samples from psoriasis patients in the GSE117468 [17] dataset were included for the analysis of clinical significance. After downloading the raw probe-level expression data, we performed background correction and quantile normalization using the robust multi-array average (RMA) algorithm implemented in the R package oligo (v1.74.0). Subsequently, probe IDs were mapped to gene symbols based on annotation information for the GPL570 platform, which was retrieved from the hgu133plus2.db (v3.13.0). For genes corresponding to multiple probes, the mean expression level of all associated probes was used as the final expression level for the respective gene. Thereafter, multiple datasets were integrated using the R package inSilicoMerging (v1.74.0), and batch effects were eliminated using the ComBat function from the R package sva (v3.58.0) [18]. Moreover, the dataset of immune-related genes comprises 782 genes from 28 peripheral immune cells derived from the previous study (See **S1 Table**) [19].

**Table 1:** Sources and sample information of datasets in this study.

Application	Dataset	Platform	Included skin samples	
			lesional (LS)	Non-lesional (NL)
	GSE30999	GPL570	85	85
Training set	GSE106992	GPL570	67	67
	GSE14905	GPL570	28	33
Validation set	GSE78097	GPL570	27	6
Clinical analysis	GSE117468	GPL570	216	–

### Differential gene analysis

The R package limma (v3.66.0) was employed to conduct differential analysis between the LS groups and the NL group. The volcano and bidirectional clustering heat maps were constructed and visualized with the thresholds |log of fold change| > 1.5 and adjusted *P* value < 0.05.

### WGCNA

The merged training datasets were subjected to analysis using the R package WGCNA (v1.74.0) for all genes. Initially, the median absolute deviation (MAD) of each gene was calculated, and the top 50% of genes with the smallest MAD were excluded. Subsequently, outlier genes and samples were removed, and a scale-free co-expression network was constructed using WGCNA. The module eigenvalue (ME) value for each module was calculated. Moreover, the correlation coefficient and *p* value between the ME value and the phenotype of clinical traits (LS vs. NL) were calculated. Modules with a *p* value of less than 0.05 were considered to be key modules associated with LS. The module membership (MM) of a gene is defined as the correlation between the gene and its respective module. Gene significance (GS) refers to the relationship between a gene and a clinical trait. In accordance with the established criteria (|MM| > 0.8 and |GS| > 0.1), the hub genes with high connectivity within the clinically significant module were identified.

### Functional enrichment analysis

The immune-key gene datasets were analyzed for overlap with hub gene sets derived from WGCNA and differential genes. Gene Ontology (GO) and Kyoto Encyclopedia of Genes and Genomes (KEGG) enrichment analysis were conducted on the immune-key genes using the R package clusterProfiler (v4.18.4). Furthermore, the ten most significant GO terms and KEGG pathways are illustrated in a bubble chart.

### LASSO and PPI network

In this study, immune-related hub genes were obtained through an overlap analysis of LASSO-screened genes with PPI network genes. Specifically, we used the R package glmnet (v4.1.10) for further downscaling analysis of immunity key genes using the LASSO. The PPI analysis of immune-key genes was conducted through the STRING database (https://cn.string-db.org/), where the minimum required interaction score was set to 0.15 (low confidence), and the results were subsequently visualized. The core network genes were selected using the molecular complex detection (MCODE) and the maximum clique centrality (MCC) algorithm of CytoHubba plugin within Cytoscape software (v3.10.1).

### Immune infiltration analysis

CIBERSORTx (https://CIBERSORTx.stanford.edu/) was used to quantify the relative abundances of 22 immune cell subsets based on linear support vector regression [20,21]. Briefly, the preprocessed expression matrix was uploaded to the CIBERSORTx web platform, with the “2. Impute Cell Fractions” module selected for analysis. The analysis was performed in “Custom” mode, and the LM22 signature matrix was adopted for deconvolution. The relative mode was selected for the run, with 500 permutations set for significance analysis; all remaining parameters were used with default settings. Only samples with a deconvolution *P* < 0.05 were retained for subsequent analysis and the Wilcoxon test was employed to screen for significantly immune cells between LS and NL groups. Furthermore, the correlation between immuno-related hub genes and differential immune cells was assessed.

### Single-gene GSEA enrichment analysis

For GSEA, we obtained the GSEA software (v3.0) from the website (http://software.broadinstitute.org/gsea/index.jsp), divided the samples into LS group and NL group, and downloaded the kegg.v7.4 subset from the Molecular Signatures Database (http://www.gsea-msigdb.org/gsea/downloads.jsp). The associated pathways and molecular mechanisms were evaluated based on gene expression profiles and phenotypic group. The minimum gene set was set to 5, the maximum gene set was set to 5000, *P* value < 0.05 and false discovery rate (FDR) < 0.25 were considered statistically significant.

### ROC curve analysis and logistic regression model

In this study, ROC and area under the ROC curve (AUC) were used to assess the discriminatory ability of the immune-related hub genes. The “enter” method was used to construct the logistic regression model. MedCalc software (v23.0.2) was used to perform these analyses.

### Clinical significance of immune-related hub genes in psoriasis

GSE117468 included patients with moderate to severe psoriasis treated with brodalumab (an Anti–Interleukin-17 Receptor Antibody) or placebo at 0, 4 and 12week. After excluding patients treated with placebo, we analyzed the expression levels of immune-related hub genes in correlation with psoriasis area and severity index (PASI) scores and treatment time.

### Drug-gene interaction analysis

The DGIdb contains data about drug-gene interactions for over 40,000 genes, 10,000 drugs, and 100,000 drug-gene interaction relationships across 42 drug categories and at least 49 interaction types, including inhibitors, activators, cofactors, ligands, and vaccines [22]. For drug-gene interaction analysis of immune-related hub genes, we utilized the DGIdb (https://www.dgidb.org/) as a resource to identify promising drug targets. The Cytoscape software was used to present the drug-gene Interaction network.

### Statistical analysis

The R (v4.3.0), GraphPad (v9.5.1), and MedCalc (v23.0.2) were used for data processing and visualization. Student’s t-tests or one-way analysis of variance (ANOVA) were used for statistical tests of two or more groups. Pearson’s correlation coefficient was employed to examine the strength of the relationship between the variables.

## Results

### Dataset processing and differential gene analysis

**Fig 2:**
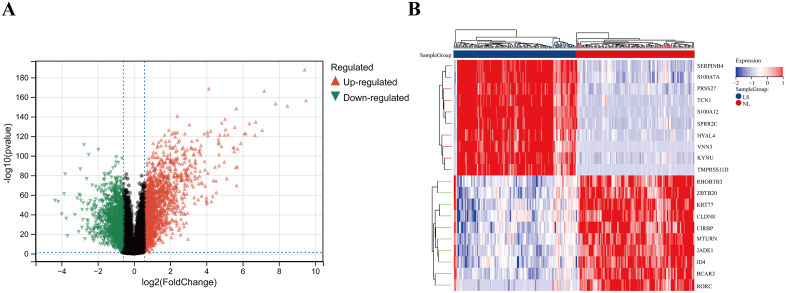
Genes differentially expressed between NL and LS group. **(A)** Volcano map of differentially expressed genes. **(B)** The heatmap of the top twenty upregulated and downregulated differentially expressed genes.

The UMAP plots and data density analyses (**see** **S1 Fig**) demonstrated the necessity of dealing with batch effects. The combined dataset comprises 365 samples and 20,547 genes. A total of 3,281 differential genes were identified through the application of filtering criteria (**see** **S2 Table**), as illustrated in the volcano map (**Fig 2A**). The heat map (**Fig 2B**) showed the top 20 differential genes.

### Identification of WGCNA modules

**Fig 3:**
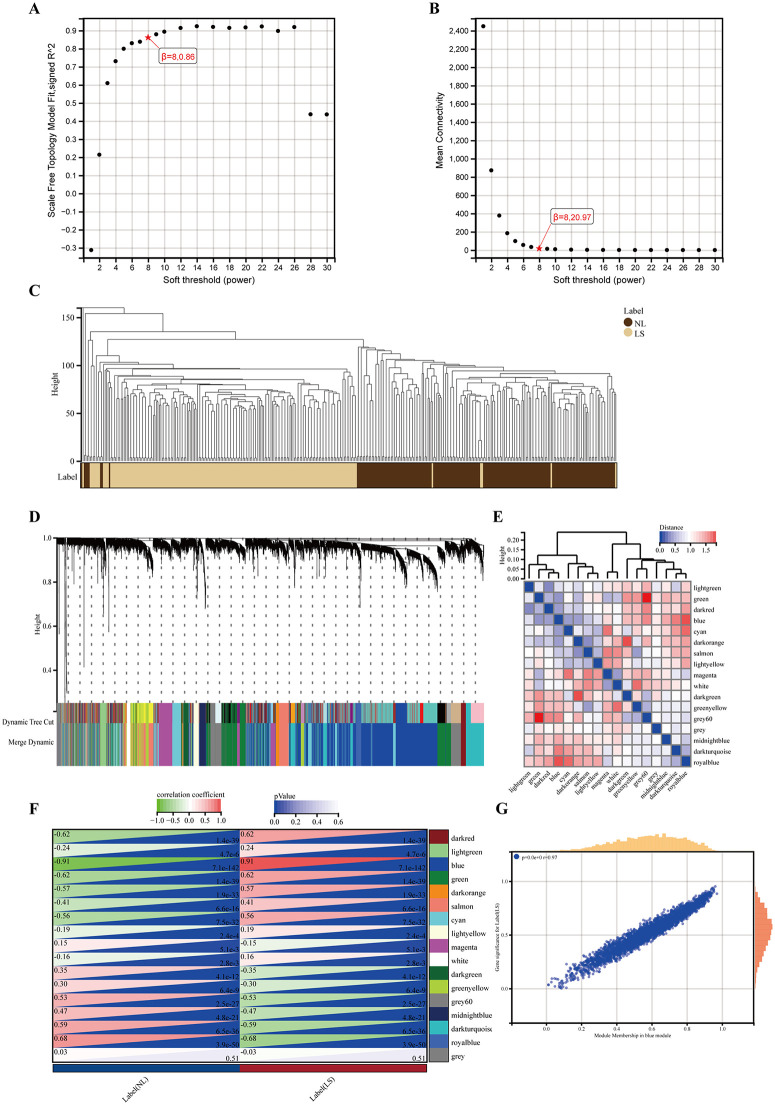
WGCNA analysis of GSE30999, GSE106992, and GSE14905. **(A)** Fit index of the corresponding scale free topology models for different soft thresholds. **(B)** Mean connectivity for different soft thresholds. **(C)** Dendrogram and trait heatmap of all sample. **(D)** Cluster dendrogram of genes. Each color represented a module, and the gray module included the genes that could not be classified into any module. **(E)** Heatmap of feature vector clustering between different modules. **(F)** Heatmap of module-phenotype correlations. **(G)** Scatterplot of the correlation between GS and MM in the blue module.

We conducted the WGCNA analysis after removing and filtering samples. Scale independence was achieved at 0.86, with an average connectivity of 20.97 and a soft threshold parameter of 8.0 (**Fig 3A and 3B**). To categorise genes with analogous expression profiles into gene modules, average linkage hierarchical clustering was performed in accordance with the TOM-based dissimilarity measure (**Fig 3C**). Modules with a divergence of less than 25% and fewer than 50 genes were merged into larger modules. Ultimately, 17 co-expression modules were identified (**Fig 3D**), along with module feature vector clustering (**Fig 3E**). Subsequently, a correlation analysis was conducted between each module and the clinical traits. The blue module exhibited the highest positive correlation with LS (**Fig 3F**). The blue module, comprising 3,834 genes, was identified as the clinically significant module for further analysis. The correlation analysis between the MM and GS (**Fig 3G**) revealed a strong association between the genes of the blue module and phenotypes (r = 0.97, *p* < 0.01). The cut-off criteria identified 513 genes with high connectivity in the blue module as WGCNA hub genes (**see** **S3 Table**).

### Screening and functional enrichment analysis of immune-key genes

**Fig 4:**
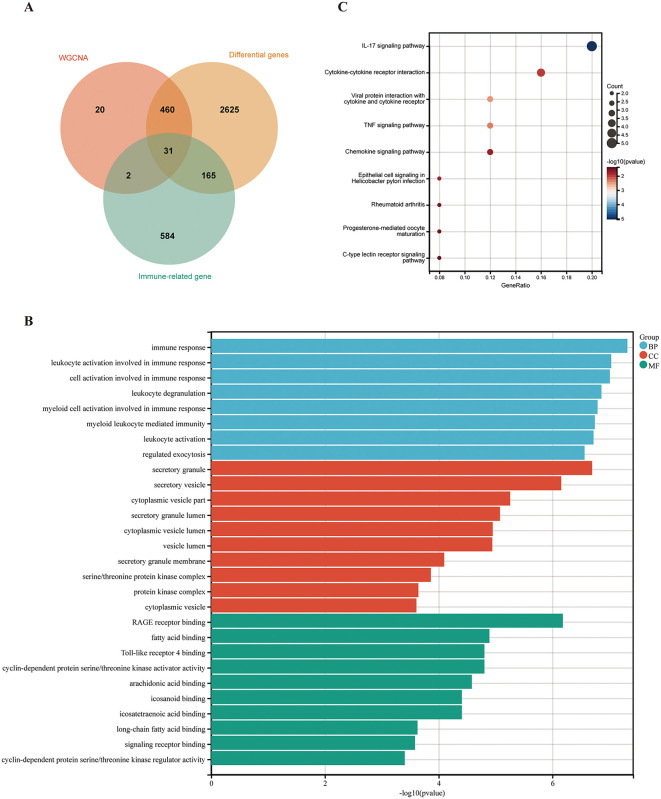
Identification and enrichment analysis of immune-key genes. **(A)** Venn diagram for three screening methods. **(B)** GO enrichment. **(C)** KEGG signaling pathway.

Following the overlapping of differential genes, blue module hub genes, and immune-related genes, 31 immune-key genes related to psoriasis were identified (**see** **S4 Table**), as illustrated in **Fig 4A**. Subsequently, a functional enrichment analysis was conducted on the aforementioned immune-key genes. The results of the GO enrichment analysis indicated that the majority of these genes were significantly enriched in biological processes associated with immune responses, including leukocyte and myeloid activation (**Fig 4B**). Furthermore, the cellular component analysis indicated that these genes were involved in secretion activities. KEGG pathway enrichment analysis demonstrated that these genes were predominantly involved in the IL-17 signaling pathway, the TNF-α signaling pathway, and the cytokine-cytokine receptor interaction pathway (**Fig 4C**), which is closely associated with the occurrence and development of psoriasis.

### Identification of immune-related hub genes in psoriasis

**Fig 5:**
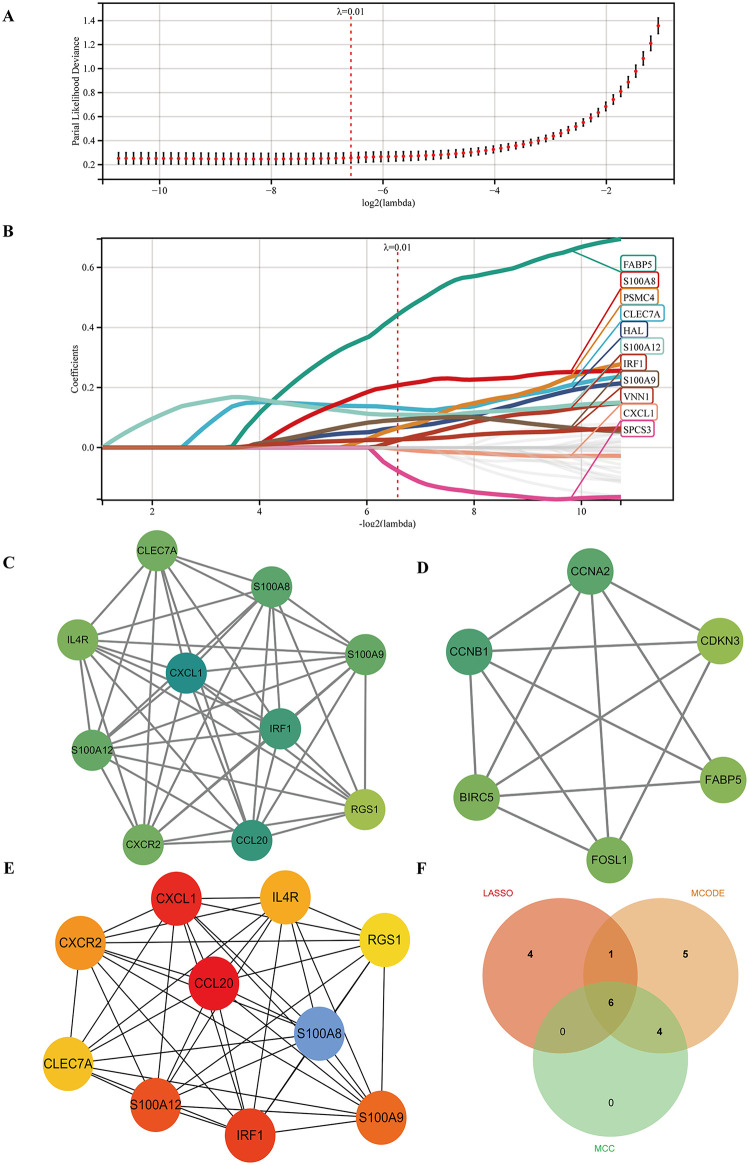
Identification of immune-related hub genes. **(A)** The partial likelihood deviance under per-fold cross-validation for different λ, the dashed line corresponding to the horizontal coordinate point is the optimal λ value. **(B)** LASSO weight coefficient profile with 1 non-zero coefficient feature selected at the optimal value of λ. (C) and **(D)** Two clusters comprising 17 central genes by the MCODE algorithm. **(E)** The top 10 genes ranked by the MCC algorithm of CytoHubba. **(F)** Venn diagram for different methods screening hub genes.

To identify immune-related hub genes for psoriasis, a total of 31 immune-key genes were subjected to LASSO model. In accordance with the value of the optimal lambda criteria (λ = 0.01), 11 central genes *(CLEC7A, CXCL1, FABP5, HAL, IRF1, PSMC4, S100A12, S100A8, S100A9, SPCS3*, and *VNN1*) were identified to have non-zero regression coefficients (**Fig 5A and 5B**). Subsequently, a PPI network was constructed using the STRING database. Moreover, the Cytoscape software was employed to identify two clusters comprising 17 central genes through the MCODE algorithm (**Fig 5C and 5D**) and the top 10 genes ranked by the MCC algorithm of CytoHubba (**Fig 5E**), respectively. Ultimately, six genes were identified as immune-related hub genes in psoriasis: *CLEC7A, CXCL1, IRF1, S100A12, S100A8*, and *S100A9* (**Fig 5F**).

### Correlation analysis and single-gene GSEA enrichment analysis

**Fig 6:**
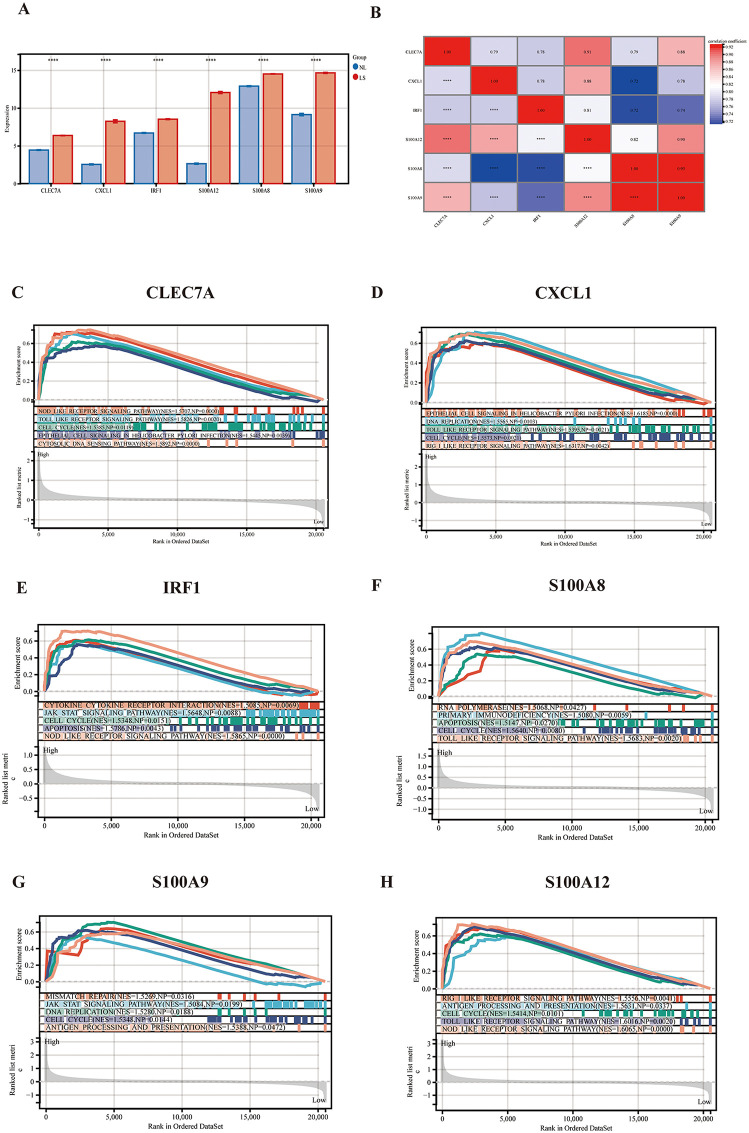
Correlation analysis and GSEA enrichment analysis of immune-related hub genes. **(A)** Bar graph of expression levels of 6 hub genes. **(B)** Correlation analysis between hub gene. GSEA analysis of *CLEC7A* **(C)**, *CXCL1* **(D)**, *IRF1* **(E)**, *S100A8* **(F)**, *S100A9* **(G)**, and *S100A12* **(H)**.

We compared the expression levels of immune-related hub genes in the LS and NL groups. The bar graphs show that the expression level of each immune-related hub genes was markedly elevated in the LS group (**Fig 6A**). Furthermore, correlation analyses revealed strong correlations (r ≥ 0.9) between *S100A12* and *CLEC7A, S100A9* and *S100A8*, and *S100A9* and *S100A12* (**Fig 6B**). GSEA indicated significant associations of these genes with the cell cycle, apoptosis, cytokine-cytokine receptor interaction, and Toll-like receptor signaling pathways, with normalized enrichment score (NES) values >1.5, nominal *p* (NP) values <0.05, and false discovery rate (FDR) q values <0.25, suggesting their roles in psoriasis (Figs 6C**-**6H).

### ROC curve analysis of immune-related hub genes

**Fig 7:**
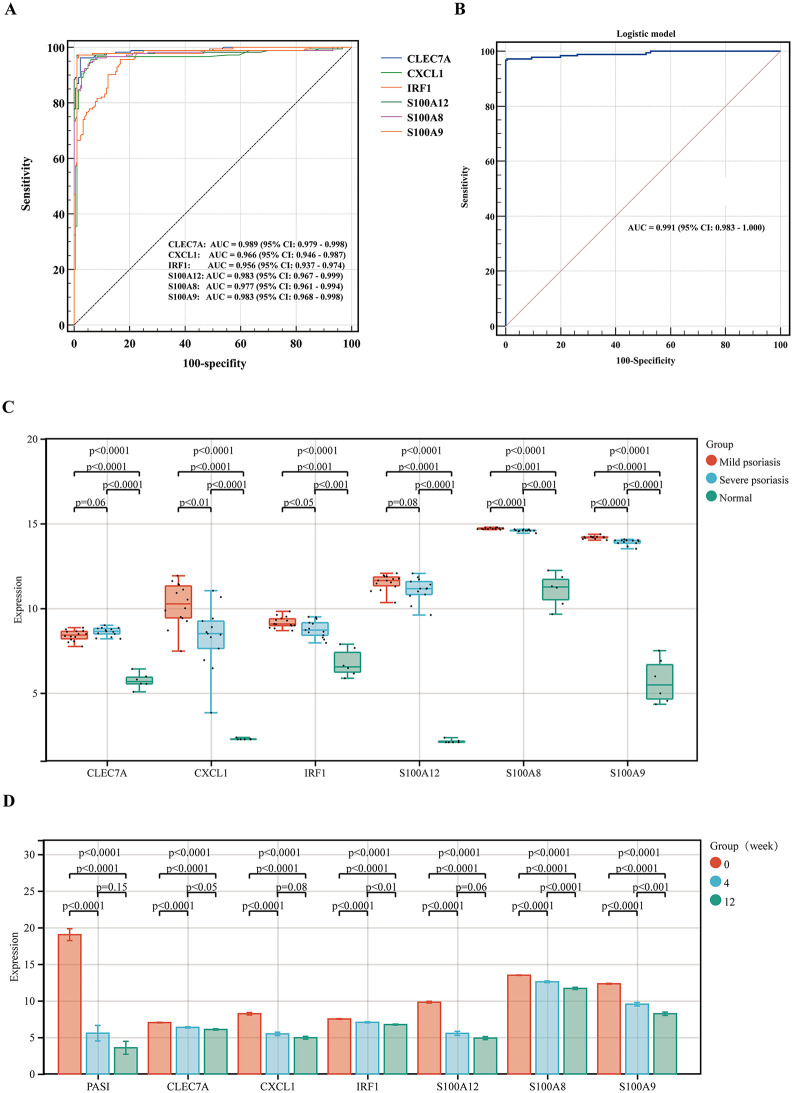
The exploratory discriminatory ability of hub genes and expression level validation. **(A)** ROC curve analysis of six hub genes. **(B)** ROC curve analysis of logistic regression models. **(C)** Expression levels of immune-related hub genes in GSE78097. **(D)** Expression levels of immune-related hub genes in GSE117468.

To evaluate the exploratory within-sample discrimination of the above genes, ROC curves were analyzed using Medcalc software, and the AUC values of different indicators were compared. The AUC values of each immune-related hub gene (*CLEC7A, CXCL1, IRF1, S100A12, S100A8,* and *S100A9*) were 0.989, 0.966, 0.956, 0.983, 0.977, and 0.983, respectively (**Fig 7A**). A statistical comparison of the AUC values indicates that *CLEC7A*, *S100A12*, and *S100A9* exhibit discriminatory ability within the analyzed cohorts. Furthermore, a logistic regression algorithm was utilized to construct a multi-gene prediction model. As shown in **Fig 7B**, the AUC value of the six-gene-combined logistic model was 0.991 (*p* ＜ 0.001).

### Validation of immune-related hub genes

The expression levels of immune-related hub genes were verified in GSE78097. **Fig 7C** shows that the expression of all six hub genes was significantly upregulated. Interestingly, the expression levels of *CXCL1, IRF1, S100A8*, and *S100A9* were significantly higher in patients with severe psoriasis than in patients with moderate psoriasis.

### Clinical significance of immune-related hub genes in psoriasis

**Fig 8:**
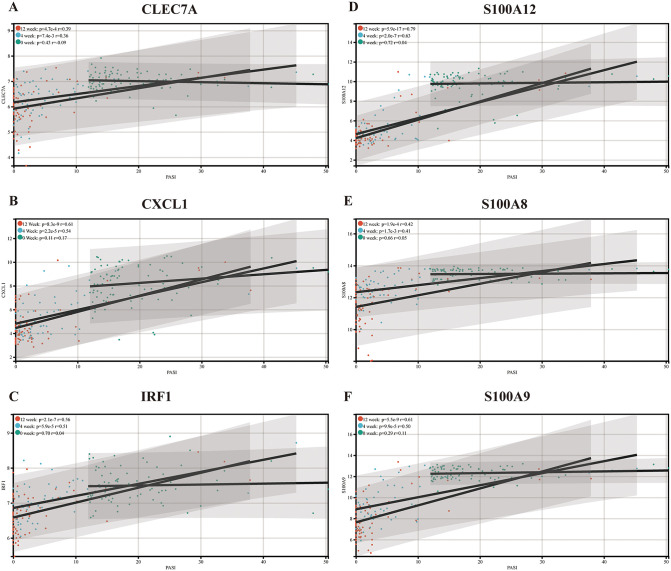
Correlation analysis between PASI score and expression levels of *CLEC7A.* (A), *CXCL1* (B), *IRF1* (C), *S100A12* (D), *S100A8* (E), and *S100A9* (F) at different treatment times.

**Fig 9:**
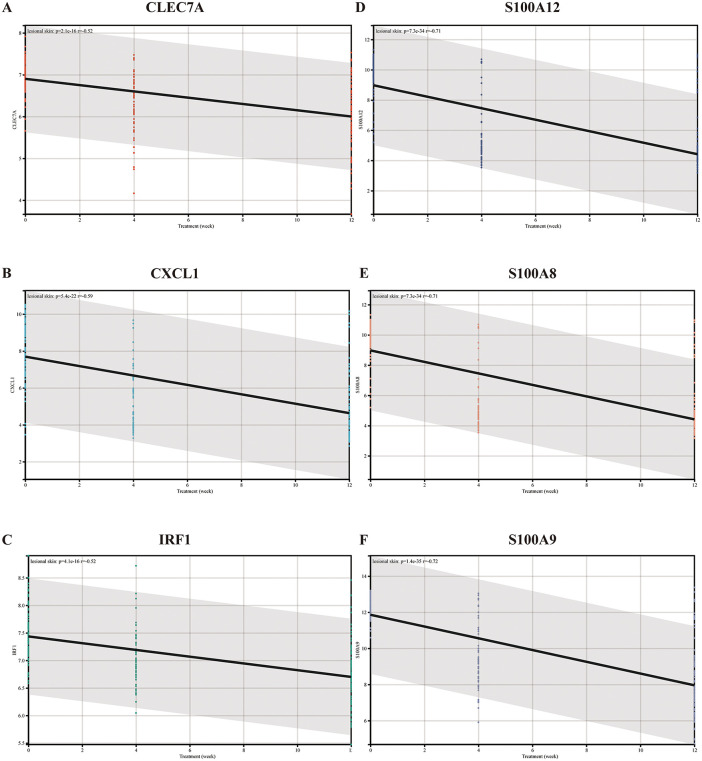
Correlation analysis between treatment length and expression levels of *CLEC7A* (A), *CXCL1* (B), *IRF1* (C), *S100A12* (D), *S100A8* (E), and *S100A9* (F).

To confirm the value of immune-related hub genes for clinical applications, we used microarray datasets of skin biopsies from psoriasis patients treated with brodalumab or placebo. The PASI scores and expression level of the hub genes were significantly decreased at 12 weeks of treatment (Fig 7D). Although no correlation was observed between PASI and expression levels of hub genes before treatment, a significant positive correlation was evident between the two variables following treatment (Fig 8A-8F). Meanwhile, the correlation analysis demonstrated that there was a negative correlation between the expression levels of hub genes and treatment time (Fig 9A-9F).

### Immune infiltration analysis

**Fig 10:**
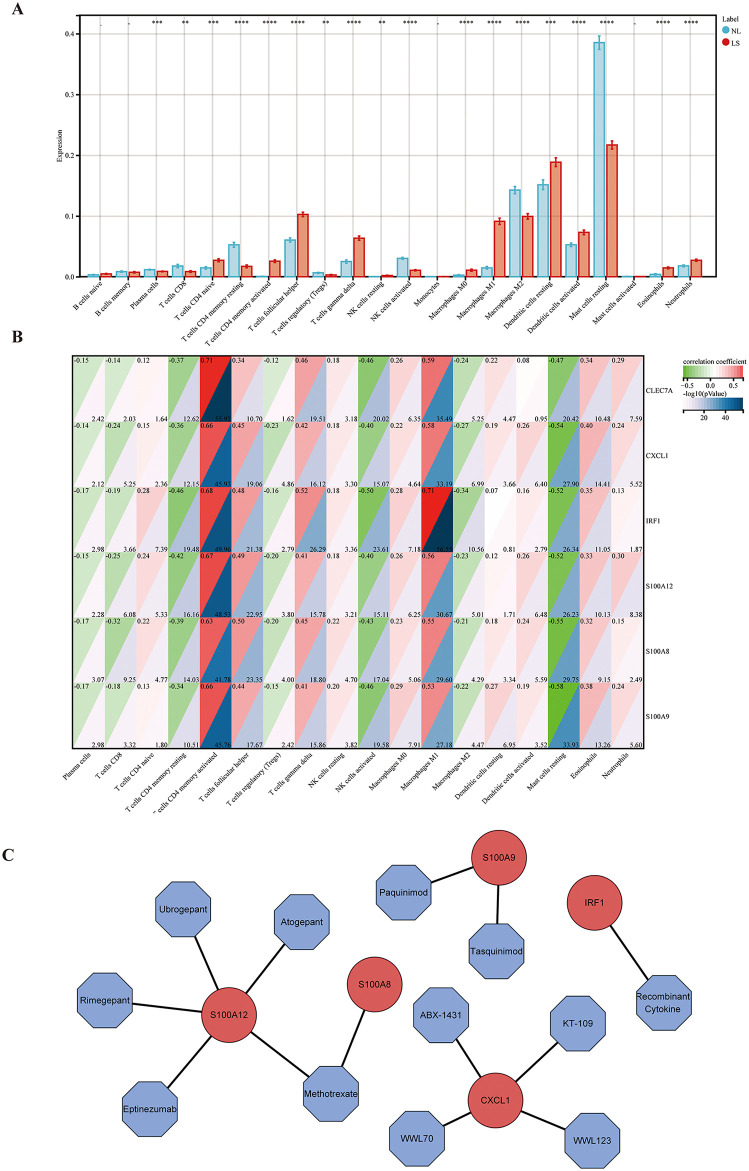
Immune infiltration analysis and drug prediction. **(A)** The boxplot of the differences in immune cell infiltration between LS and NL samples. **(B)** The correlation plot of the relationships among immune cells and the six immune-related hub genes. **(C)** Drug prediction network diagram, red circles represent hub genes and blue polygons represent potential drugs. ^-^, *p* > 0.05, *p < 0.05, **p < 0.01, ***p < 0.001, ****p < 0.0001.

Significant discrepancies in immune cell composition were identified between LS and NL samples using the CIBERSORTx (**Fig 10A**). The results of immune cell infiltration showed that the percentage of naive CD4 T cells, activated memory CD4 T cells, gamma delta T cells, resting NK cells, M0 macrophages, M1 macrophages, dendritic cells, eosinophils, and neutrophils in the LS group was significantly higher than that of the NL group, Conversely, plasma cells, CD8 T cells, resting memory CD4 T cells, Tregs, activated NK cells, M2 macrophages, and resting mast cells were significantly lower than in the NL group. A correlation analysis was performed and showed a strong association between the hub genes and immune cells (**Fig 10B**). Specifically, we found that the six immune-related hub genes were highly positively correlated with activated memory CD4 T cells and M1 macrophages.

### Gene-drug analysis

There are 5 drugs that interact with *S100A12*, 4 drugs that interact with *CXCL1*, 2 drugs that interact with *S100A9*, 1 drug that interacts with *S100A8,* and 1 drug that interacts with *IRF1* (**Fig 10C**). Although the gene-drug interaction analysis derived from DGIdb represents only an exploratory association without experimental or clinical validation, these findings provide novel insights and potential strategies for the future prevention and clinical treatment of psoriasis.

## Discussion

Psoriasis is a common, relapsing, chronic inflammatory skin disease that places a burden on individuals, healthcare systems and societies worldwide [23–25]. Despite the notable advancement in the efficacy of biologics in managing symptoms, the treatment of psoriasis still encounters challenges, including a high relapse rate and the difficulty in identifying early exacerbations [26]. With the advancement of genomic technologies, we can comprehensively investigate transcriptomic alterations under psoriasis and identify potential hub genes via bioinformatic analysis. For instance, Liu et al. [27]. identified *S100A8/S100A9* as hub genes in psoriasis but failed to perform cross-dataset validation to confirm the stability of these genes. Wang et al. [28]. analyzed *ARG1* and *CXCL2* as potential biomarkers in the context of psoriasis-related immune dysregulation, yet did not explore their association with treatment outcomes. In contrast, our study integrated five independent microarray datasets of psoriasis for the systematic screening and validation of immune hub genes. More importantly, we further established a novel link between these immune hub genes and treatment response (e.g., response to anti-TNF-α therapy), thereby revealing their potential as predictive biomarkers for therapeutic efficacy.

In our work, a total of 31 immune-related key genes were identified through differential gene analysis, WGCNA, and an immune gene set, which were implicated in a variety of immune responses, predominantly enriched in the IL-17 signaling pathway, cytokine interactions, and the TNF signaling pathway. These findings are consistent with previous researches in psoriasis [29–31], suggesting that immune and inflammatory responses play a key role in the pathogenesis of psoriasis. LASSO is a powerful machine learning algorithm, especially for feature selection and model simplification, and is used to screen variables in bioinformatics analysis [32,33]. A PPI network is mainly used to study the interactions between proteins in organisms, with the aim of revealing information about protein function, cell signaling, and disease occurrence mechanisms [34]. In this study, LASSO and the PPI network were employed to identify six immune-related hub genes, including *CLEC7A, CXCL1, IRF1, S100A12, S100A8*, and *S100A9*, all of which exhibited high AUC values (AUC > 0.9). It should be noted that since these analyses were based on the dataset used for gene selection, the discriminatory ability of the identified hub genes may be overestimated without independent external validation. Furthermore, the identification of hub genes based on network analysis is purely exploratory in nature. Nevertheless, the high network centrality of these hub genes suggests they are promising candidates that warrant prioritization for follow-up studies, though their mechanistic or causal significance in the occurrence and progression of psoriasis remains to be established. Additionally, correlation analysis revealed that these genes are closely associated with the severity of psoriasis and the therapeutic response to biological agents.

Moreover, we conducted an immune infiltration analysis by CIBERSORTx, a tool to deconvolve gene expression profiles and estimate the proportions of immune cell subsets. The results show that compared with the NL group, naive CD4 T cells, activated memory CD4 T cells, gamma delta T cells, resting NK cells, M0 macrophages, M1 macrophages, dendritic cells, eosinophils, and neutrophils infiltrated more in the LS group, while plasma cells, CD8 T cells, resting memory CD4 T cells, Tregs, activated NK cells, M2 macrophages, and resting mast cells infiltrated less. Notably, CIBERSORTx was established based on a peripheral blood immune cell reference signature and is therefore not fully optimized for skin tissue [21]. Abundant stromal cells (e.g., keratinocytes, fibroblasts) in skin may interfere with deconvolution and introduce bias [35]. This method only provides relative immune cell proportions rather than absolute counts, and has limited discrimination for rare or highly similar immune cell subsets. Despite these limitations, our analysis offers a preliminary profile of the immune microenvironment in psoriasis.

*CLEC7A* (also known as dectin-1, C-type lectin domain containing 7A) is a C-type lectin receptor belonging to a family of pattern-recognition receptors that play a role primarily in antifungal immunity. The expression of dectin-1 is upregulated in psoriatic lesions [36]. Some studies showed that IL-17A and TNF-α may promote the proliferation of keratinocytes within psoriatic lesions by inducing Dectin-1, suggesting that dectin-1 may be a potential therapeutic target for the treatment of psoriasis [37]. Acting as a chemokine, *CXCL1*(C-X-C motif chemokine ligand 1) induces cell migration, particularly of leukocytes, by binding to its receptor *CXCR2*, which is increased in keratinocytes from psoriasis patients [38]. Our study demonstrated that the expression level of *CXCL1* was increased and further reduced in response to treatment. In addition, our correlation analysis revealed a positive correlation between *CXCL1* and neutrophils and CD4 memory T cells. The protein encoded by the *IRF1* (interferon regulatory factor 1) gene is a transcriptional regulator and tumor suppressor that acts as an activator of genes involved in the innate and acquired immune response [39]. It has been reported that *IRF1* acts as a transcription factor and binds to the promoter region of STAT1 to facilitate macrophage M1 polarization to promote inflammatory lung injury [40]. Our study’s results support the aforementioned conclusions. GSEA analysis shows that *IRF1* is involved in cytokine interactions as well as the JAK/STAT1 signaling pathway. Moreover, the immune infiltration analysis demonstrated an increase in M1 macrophages, which play a pro-inflammatory role, and a decrease in M2 macrophages, which contribute to anti-inflammatory responses and tissue repair. A notable correlation was observed between *IRF1* and M1 macrophages (r = 0.71). In a word, *IRF1* plays a role in the pro-inflammatory response against psoriasis, suggesting its potential as a targeted therapeutic agent [41].

The S100 calcium binding protein family is a highly conserved family of Ca² ⁺ -binding proteins comprising at least 25 members, which play a role in a number of biological processes [42–44]. In the field of dermatology, the S100 family is an important multifunctional player in inflammatory skin diseases, and a number of S100 proteins, including *S100A2*, *S100A7*, *S100A8*, *S100A9*, *S100A12*, and *S100A15* [45], have been identified as being closely associated with the progression of psoriasis. In our work, *S100A8*, *S100A9*, and *S100A12* were also identified as immune-related hub genes and exhibited a statistically significant correlation with the immune cell. *S100A8/A9* is a heterodimer of *S100A8* and *S100A9*, also known as calprotectin, which has been observed to be elevated in both skin lesions and plasma samples from patients with psoriasis [42,46–48], indicating that it may serve as a biomarker of psoriasis activity. *S100A8* and *S100A9* may serve as key mediators in psoriasis pathogenesis by promoting IL-17 synthesis, modulating the proliferation and differentiation of keratinocytes, and triggering inflammatory responses via binding to Toll-like receptor 4 (TLR4) [45,49]. In accordance with the findings of previous studies, the GSEA enrichment analysis demonstrated that *S100A8/S100A9* were involved in cell proliferation, apoptosis, and Toll-like receptor signaling pathways. These findings identify potential new targets for the treatment of psoriasis and underscore the necessity for further investigation into the role of *S100A8/S100A9* in the pathological process of psoriasis. *S100A12* is a pro-inflammatory protein that is expressed in keratinocytes, and its level is positively correlated with the severity of psoriasis [50], which is similar to our study. The interaction between *S100A12* and the receptor for advanced glycation end products domain enhances the inflammatory response of cells to oxidative stress, which plays a role in the pathogenesis of psoriasis. The binding of *S100A12* to TLR4 activates the TLR4 signaling pathway and increases the expression of pro-inflammatory cytokines [51]. Interestingly, our study found the *S100A8, S100A9* and *S100A12* showed similar expression patterns as well as a positive correlation with activated CD4 T cell infiltration. Furthermore, genes-drugs analysis showed that the S100 family of proteins may be employed as drug targets for the treatment of psoriasis.

In summary, several studies have reported hub genes associated with psoriasis through biometric analyses [52], but the identification of immune-related hub genes and their association with severity of skin lesions and treatment of biologics have been rarely reported. In this work, we not only validated the upregulation of the six immune-related hub genes in an independent psoriasis dataset but also found that these genes were more highly expressed in severe psoriatic skin lesions. In addition, there was a strong correlation between hub gene expression and PASI after treatment, and their expression levels were significantly reduced after treatment with biologics, suggesting that the above gene expression patterns warrant further investigation to determine if they could eventually inform clinical monitoring. Most hub genes were previously reported to be expressed in keratinocytes, but we found correlations with activated CD4 T cells and macrophages, and gene-drug analysis showed that some of the genes had sufficient data on drug response, and in the future, the development of targeted drugs against these genes may have some potential. This study has some limitations. Firstly, in the future, it is essential to validate the expression levels of hub genes on the individual cell through single-cell sequencing data. In the present study, using multiple dimensionality reduction and network analysis approaches, we identified and proposed putative key genes that may be implicated in psoriasis. Nevertheless, experimental evidence is currently insufficient to substantiate the correlative and causal relationships of these genes. Furthermore, the functional mechanisms of these hub genes and their interactions with immune cells remain to be further explored. Crucially, as this study relies on purely in silico analyses, future ‘wet lab’ validation—such as reverse transcription-quantitative polymerase chain reaction (RT-qPCR) or immunohistochemistry (IHC) in local, independent patient cohorts—is essential to confirm these findings as definitive clinical biomarkers.

## Conclusion

The study identified six immune-related hub genes that were closely related to the severity of psoriasis and the effectiveness of biological treatments. In addition, there was a significant difference in immune cell infiltration between the LS and NL groups, with strong positive correlations between activated memory CD4 T cells and M1 macrophages and six immune-related hub genes.

## Supporting information

S1 Table Immune-related genes comprise 782 genes from 28 peripheral immune cells. (DOCX)

S1 Fig Analysis of de-batch effect. (A) Density diagram and UMAP plot (B) of the dataset before the de-batch effect. (C) Density diagram and UMAP plot (D) of the dataset after the de-batch effect. (E) Bar chart of quantitative assessment of variance of before and after de-batch effect.
(TIF)

S2 Table 3,281 differential genes were identified. (XLSX)

S3 Table 513 genes with high connectivity in the blue module as WGCNA hub genes. (XLSX)

S4 Table 31 immune-key genes related to psoriasis. (XLSX)
