# Supplementary figures and images for: Identification and clinical implications of immune-related hub genes in psoriasis
Source: PLoS One. 2026 Apr 20;21(4):e0347536. doi: 10.1371/journal.pone.0347536 (PMC13095007; doi:10.1371/journal.pone.0347536)

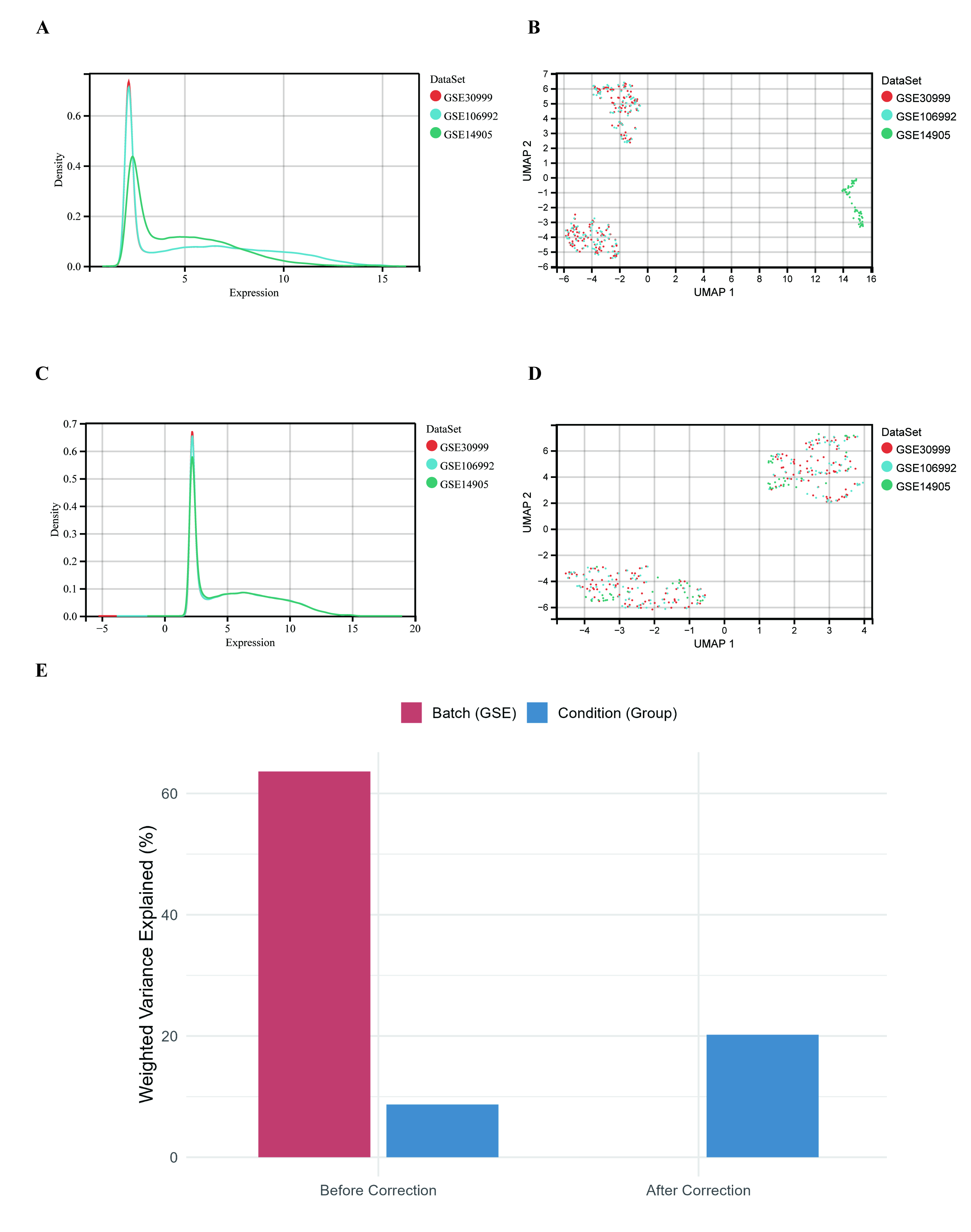

Supplement: S1 Fig — (A) Density diagram and UMAP plot (B) of the dataset before the de-batch effect. (C) Density diagram and UMAP plot (D) of the dataset after the de-batch effect. (E) Bar chart of quantitative assessment of variance of before and after de-batch effect. (TIF) [file pone.0347536.s002.tif]
